# Eliciting Insights From Chat Logs of the 25X5 Symposium to Reduce Documentation Burden: Novel Application of Topic Modeling

**DOI:** 10.2196/45645

**Published:** 2023-05-17

**Authors:** Amanda J Moy, Jennifer Withall, Mollie Hobensack, Rachel Yeji Lee, Deborah R Levy, Sarah C Rossetti, S Trent Rosenbloom, Kevin Johnson, Kenrick Cato

**Affiliations:** 1 Department of Biomedical Informatics Columbia University New York, NY United States; 2 School of Nursing Columbia University New York, NY United States; 3 School of Medicine Yale University New Haven, CT United States; 4 Veteran’s Affairs Connecticut Health Care System Pain, Research, Informatics, Multi-morbidities Education Center West Haven, CT United States; 5 Department of Biomedical Informatics Vanderbilt University Nashville, TN United States; 6 Department of Biostatistics, Epidemiology and Informatics University of Pennsylvania Philadelphia, PA United States; 7 Department of Computer and Information Science University of Pennsylvania Philadelphia, PA United States; 8 Department of Emergency Medicine Columbia University Irving Medical Center New York, NY United States; 9 Department of Biomedical and Health Informatics Children’s Hospital of Philadelphia Philadelphia, PA United States

**Keywords:** topic modeling, content analysis, online chat, virtual conference, documentation burden, burnout, physicians, nurses, policy, symposium, chat bot

## Abstract

**Background:**

Addressing clinician documentation burden through “targeted solutions” is a growing priority for many organizations ranging from government and academia to industry. Between January and February 2021, the 25 by 5: Symposium to Reduce Documentation Burden on US Clinicians by 75% (25X5 Symposium) convened across 2 weekly 2-hour sessions among experts and stakeholders to generate actionable goals for reducing clinician documentation over the next 5 years. Throughout this web-based symposium, we passively collected attendees’ contributions to a chat functionality—with their knowledge that the content would be deidentified and made publicly available. This presented a novel opportunity to synthesize and understand participants’ perceptions and interests from chat messages. We performed a content analysis of 25X5 Symposium chat logs to identify themes about reducing clinician documentation burden.

**Objective:**

The objective of this study was to explore unstructured chat log content from the web-based 25X5 Symposium to elicit latent insights on clinician documentation burden among clinicians, health care leaders, and other stakeholders using topic modeling.

**Methods:**

Across the 6 sessions, we captured 1787 messages among 167 unique chat participants cumulatively; 14 were private messages not included in the analysis. We implemented a latent Dirichlet allocation (LDA) topic model on the aggregated dataset to identify clinician documentation burden topics mentioned in the chat logs. Coherence scores and manual examination informed optimal model selection. Next, 5 domain experts independently and qualitatively assigned descriptive labels to model-identified topics and classified them into higher-level categories, which were finalized through a panel consensus.

**Results:**

We uncovered ten topics using the LDA model: (1) *determining data and documentation needs* (422/1773, 23.8%); (2) *collectively reassessing documentation requirements in electronic health records* (EHRs) (252/1773, 14.2%); (3) *focusing documentation on patient narrative* (162/1773, 9.1%); (4) *documentation that adds value* (147/1773, 8.3%); (5) *regulatory impact on clinician burden* (142/1773, 8%); (6) *improved EHR user interface and design* (128/1773, 7.2%); (7) *addressing poor usability* (122/1773, 6.9%); (8) *sharing 25X5 Symposium resources* (122/1773, 6.9%); (9) *capturing data related to clinician practice* (113/1773, 6.4%); and (10) *the role of quality measures and technology in burnout* (110/1773, 6.2%). Among these 10 topics, 5 high-level categories emerged: *consensus building* (821/1773, 46.3%), *burden sources* (365/1773, 20.6%), *EHR design* (250/1773, 14.1%), *patient-centered care* (162/1773, 9.1%), and *symposium comments* (122/1773, 6.9%).

**Conclusions:**

We conducted a topic modeling analysis on 25X5 Symposium multiparticipant chat logs to explore the feasibility of this novel application and elicit additional insights on clinician documentation burden among attendees. Based on the results of our LDA analysis, consensus building, burden sources, EHR design, and patient-centered care may be important themes to consider when addressing clinician documentation burden. Our findings demonstrate the value of topic modeling in discovering topics associated with clinician documentation burden using unstructured textual content. Topic modeling may be a suitable approach to examine latent themes presented in web-based symposium chat logs.

## Introduction

### Background

Developing actionable strategies to reduce clinician documentation burden is a growing priority for researchers, thought leaders, and policy makers from many organizations ranging from government and academia to industry [1-7]. Documentation burden is defined as “work that does not add value” (ie, work beyond that which is required for good clinical care) [8]. It is associated with negative sequelae such as the potential for less succinct and accurate patient records needed to communicate necessary information for care delivery; this can lead to patient safety concerns [9], added cognitive burden [10], and burnout among clinicians [11-13]. Amid increases in health care worker turnover engendered by the COVID-19 pandemic and its associated heightened workload, the need to identify “targeted solutions” to reverse growing attrition rates among clinicians—so that patient care demands are met—grows progressively dire [14]. In fact, between 25% to 40% of clinicians including nurses, advanced practice providers, and physicians anticipate leaving the field in the next 2 years [15]. Motivated by these trends, the 25 by 5: Symposium to Reduce Documentation Burden on US Clinicians by 75% (25X5 Symposium) was convened in January 2021 to foster communication, collaboration, and dissemination of best practices among various stakeholder groups and to avoid duplication of efforts and minimize cross-purposes in reducing documentation burden [16]. Over a 6-week period, experts and stakeholders gathered weekly to exchange ideas, share their experiences surrounding documentation burden, and develop a compendium of actionable short-, medium-, and long-term goals to considerably reduce clinician documentation over the next 5 years [16]. While ambitious, the objective to reduce the documentation burden to 25% of its current state was established to align the documentation load of clinicians in the United States with their international counterparts [16]. For example, studies have demonstrated that US clinicians spend 50% more time engaging with the electronic health record (EHR) than their international counterparts (eg, Canada, Northern Europe, Western Europe, etc) and approximately 25% more time working on EHRs after their shift [17]. Controlling for EHR vendor software, clinical notes among US physicians are, on average, 4 times the character length of notes authored by physicians in other nations (eg, Canada, United Kingdom, Australia, etc) [11].

The rapid and widespread transition to EHRs has made available a plethora of quantitative data for research. These data have been applied to a number of study contexts, including those examining clinician EHR actions and EHR work. However, limited qualitative research has been dedicated to understanding critical areas of interest and current trends regarding clinician documentation practices as it directly or indirectly relates to burden among those who practice in the United States [18].

As with clinical encounters, meetings and other forms of social interaction instantaneously pivoted to web-based platforms due to the onset of the COVID-19 pandemic in March 2020. The 25X5 Symposium was initially planned as an in-person event, and planners adapted to a web-based symposium platform given modifications to institutional participation guidelines. At the time, the concept of web-based symposiums remained comparatively novel [19]. Recognizing the differences in web-based settings compared to conventional in-person meetings for attendee engagement [20], the transition to web-based platforms presented the 25X5 Steering Committee with the opportunity to both expand attendee capacity and passively collect data on the social interactions that publicly transpired among attendees in the chat functionality [16]. The literature on interactive web-based environments for academic learning suggests the benefits of this type of communication include, but are not limited to, socialization and idea exchange [16,21]. Throughout the 25X5 Symposium, a synchronous chat [22] functionality was made available to all attendees, who were notified that the chat content would be deidentified and made publicly available on the 25X5 Symposium website [16].

Prior studies have demonstrated the value of analyzing chat logs to understand participants’ perceptions and interests [23]. Historically, various qualitative and quantitative methods have been applied in different settings to explore chat log content, including discourse structure analysis [24], sentiment analysis [25], and topic modeling [26]. Specifically, topic modeling is a probabilistic generative approach that identifies recurring “topics”—defined as patterns of expression or mixtures of words that frequently occur together among a collection of documents—by “analyzing the words of the original texts” [27] in an unsupervised fashion [27,28]. Typically employed for text mining and information retrieval tasks, topic modeling has been widely utilized to uncover emerging themes in free text, such as emails, lay and scientific literature, social media posts, and chat logs [26,29-31]. It has been broadly conducted in many contexts, including health care and industry, to examine didactic conversational dialogue between individuals and web-based consumer support agents [31,32]. To the best of our knowledge, no studies have used topic modeling to examine chat logs in a web-based symposium setting. Furthermore, research on clinician perceptions and attitudes regarding documentation burden has centered on qualitative interviews [33,34] and surveys [35,36], which are resource-intensive and time-consuming to conduct and may additionally encumber clinicians. Few data-driven approaches have been applied to unobtrusively extract insights on clinician documentation burden at scale. Given the volume and pace at which the chat unfolded throughout the symposium, we applied topic modeling on the 25X5 Symposium chat content in this quantitative-qualitative analysis to elicit latent insights that could be harnessed for reducing clinician documentation burden.

### Objective

The objective of this study was to explore unstructured chat log content from the web-based 25X5 Symposium using topic modeling and elicit additional insights and contextual information on reducing clinician documentation burden.

## Methods

### Data Source

The 25X5 Symposium targeted representatives from clinical settings, academia, industry, government, professional organizations, payers, and patients. The symposium was promoted through a panel presentation on documentation burden and professional networking sessions at the 2020 American Medical Information Association (AMIA) Annual Symposium. Additionally, email invitations to participate were sent to a list of key clinicians and other health care leaders identified by the 25X5 Steering Committee, which comprised clinicians, informatics experts, and health care leaders [34]. Over a 6-week period from January to February 2021, over 300 participants from 140 organizations attended the symposium, which involved a series of weekly, 2-hour web-based sessions [16]. The first 4 sessions included presentations on the following subjects: (1) current challenges in documentation content and clinician workflow; (2) existing bias that is evident in how we document in the EHR and its potential upstream and downstream effects; (3) exemplars and key successes in reducing documentation burden; and (4) novel interventions and innovations presently being developed to alleviate documentation burden. The final 2 sessions of the 25X5 Symposium summarized the entire series, presented future directions, and involved breakout work among groups of 5 to 10 attendees who jointly formulated goals and interventions. Attendance was manually recorded for the first 4 sessions. Throughout the 25X5 Symposium, attendees convened over Zoom video conferencing software (Zoom Video Communications Inc), and all audio and video content was recorded. All attendees except for presenters were placed on mute; however, a synchronous chat functionality [22] that streamed concurrently with the formal presentations was made accessible to all attendees in each of the sessions. Before each session, attendees were presented with ground rules for the symposium and informed that the chat content would be deidentified and made publicly available on the 25X5 Symposium website [16] for those interested in conducting additional analysis on the meeting. Participants were encouraged to engage in the chat respectfully and freely as they personally saw fit.

### Data Cleaning and Preprocessing

**Figure 1:**
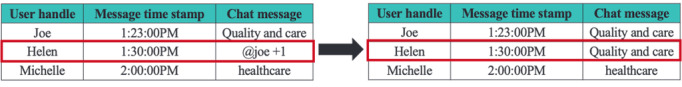
Preprocessing approach for nesting attendee chat messages referenced in a preceding message.

We concatenated Zoom chat messages that were shared publicly [16] among all attendees across the six 25X5 Symposium sessions into one analytical dataset; private messages were excluded from the analysis. The raw dataset consisted of 3 columns: message time stamp, author name (ie, user handle), as well as a textual chat message. Each chat message qualified as 1 “document” (ie, the natural grouping to understand the free text). Among chat messages that expressed agreement over a prior chat message from another attendee using the following expression, “+1” (ie, short code signifying a “thumbs up” gesture) and “[author name],” we nested the referenced attendee chat message that was most temporally adjacent to the expression (Figure 1). Based on this approach, we nested 200 messages within messages; 9 were not replaced as they expressed agreements with the synchronous presentation content.

We normalized the chat messages by removing all person names from the corpus using pattern matching with regular expressions supplemented by manual human annotation (due to Zoom user handles) and applied gensim [37] and nltk [38] libraries to eliminate stop words and common words (ie, “thank,” “thanks,” “hello,” “amen,” “lol,” “hi”), numbers, and special characters. We used WordNet (Princeton University) lemmatization [38] with part-of-speech tagging to stem words. While we examined additional (n-gram) models, we ultimately vectorized the chat log text using a bag-of-words approach given the comparatively small documentation corpus and short text length observed among chat messages [39].

### Topic Modeling and Topic Labeling

We used topic modeling to computationally explore common documentation burden topics among chat messages generated during the 25X5 Symposium. Topic models are statistical language models that are used to discover latent or unobserved semantic structures within a corpus of texts (ie, documents). Specifically, we employed a latent Dirichlet allocation (LDA) algorithm [40], a probabilistic topic model that assumes that a collection of documents is represented by a set number of topics, with each topic representing a distribution of terms (ie, words) over a fixed vocabulary and each document comprising a distribution of topics [41,42]. We evaluated model performance using coherence scores (*topic coherence [C_v]*), a measure that scores the degree of semantic similarity between co-occurring terms within a single topic, where higher topic coherence signifies the higher quality of learned topics [43]. Informed by topic coherence scores, we further explored models with 1 to 20 topics. We iterated over varying permutations for topic number (*k*), document-topic density (α), and word-topic density (β) parameters to identify the optimal model which was supplemented by domain expert examination.

Iteratively, 5 authors (AJM, JW, MH, RYL, and DRL) with wide-ranging domain expertise in medicine, health care, and informatics (ie, 3 nurse informaticists, a physician, and a data expert) independently and qualitatively assigned descriptive labels to LDA model–identified topics that best represented the distribution of keyword clusters, such as per-topic term probabilities (β) and per-document topic contribution weights based on their expert judgment. Then, the authors inductively classified structurally similar topic labels into higher-level thematic categories. All thematic categories were finalized through a panel consensus among the 5 authors.

### Statistical Analysis and Data Visualization

We generated descriptive statistics on the content of the chat log messages (ie, documents) to examine the degree of engagement among attendees using the (1) number of unique chat participants, (2) proportion of attendees who engaged in the chat, (3) frequency of chat messages, (4) average number of messages per participant, and (5) average number of words per message, stratified by session topic. We visualized overall chat log data using a word cloud of the top 100 terms (with font size proportionally representing term frequency) and calculated their relative term frequencies (ie, the frequency of which a term is used in relation to the terms used in the entire corpus [*rel_freq*]). Using bar graphs, we examined the distributions of the per-topic terms that appeared in the chat log as well as the distribution of topics stratified by session number. Lastly, we generated a t-distributed Stochastic Neighbor Embedding (t-SNE) plot [44] to visually examine topic coherence and evaluate the quality of identified topics (ie, degree of overlap among topics); t-SNE plots project high-dimensional data points onto a lower dimensional space (eg, 2D plane) so that highly complex data are human observable and interpretable. All analyses were conducted using Python 3.9.

### Ethical Considerations

The raw data are publicly available on the 25X5 Symposium website [16]. Additionally, any data that potentially represent proper nouns have been further redacted in the manuscript to preserve participant privacy.

## Results

**Figure 2:**
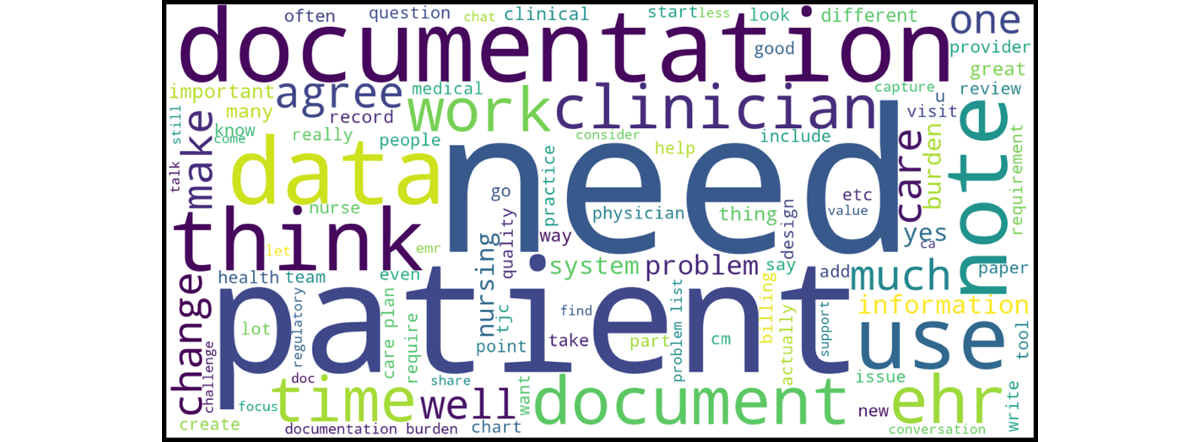
Word cloud of the top 100 frequently used terms.

Between 30.8% (52/169) and 48.8% (99/203) of participants engaged in the chat functionality at least once in each of the six 2-hour sessions (Table 1). Session 1 represented the highest volume of unique chat participants (n=99, 48.8%) and generated the most chat messages compared to the other sessions (n=470, 26.5%). We captured 1787 messages among 167 unique chat participants; 14 were private messages to the Columbia University Communications Director and were not included in the analysis. The vocabulary size of the preprocessed data was 2930. On average, chat participants shared between 2 and 6 messages per session and 11 messages across all sessions. Throughout the 6 sessions, messages were, on average, 19.7 (SD 17) words long, which represents the average length of one sentence (ie, approximately 15-20 words) in English [45]. Chat message word lengths ranged from 1 to 122 words. The top ten terms utilized in the chats were (1) “need” (*rel_freq*=1), (2) “patient” (*rel_freq*=0.94), (3) “documentation” (*rel_freq*=0.92), (4) “data” (*rel_freq*=0.77), (5) “use” (*rel_freq*=0.67), (6) “think” (*rel_freq*=0.66), (7) “note” (*rel_freq*=0.66), (8) “document” (*rel_freq*=0.62), (9) “EHR” (*rel_freq*=0.61), and (10) “time” (*rel_freq*=0.54) (Figure 2).

**Figure 3:**
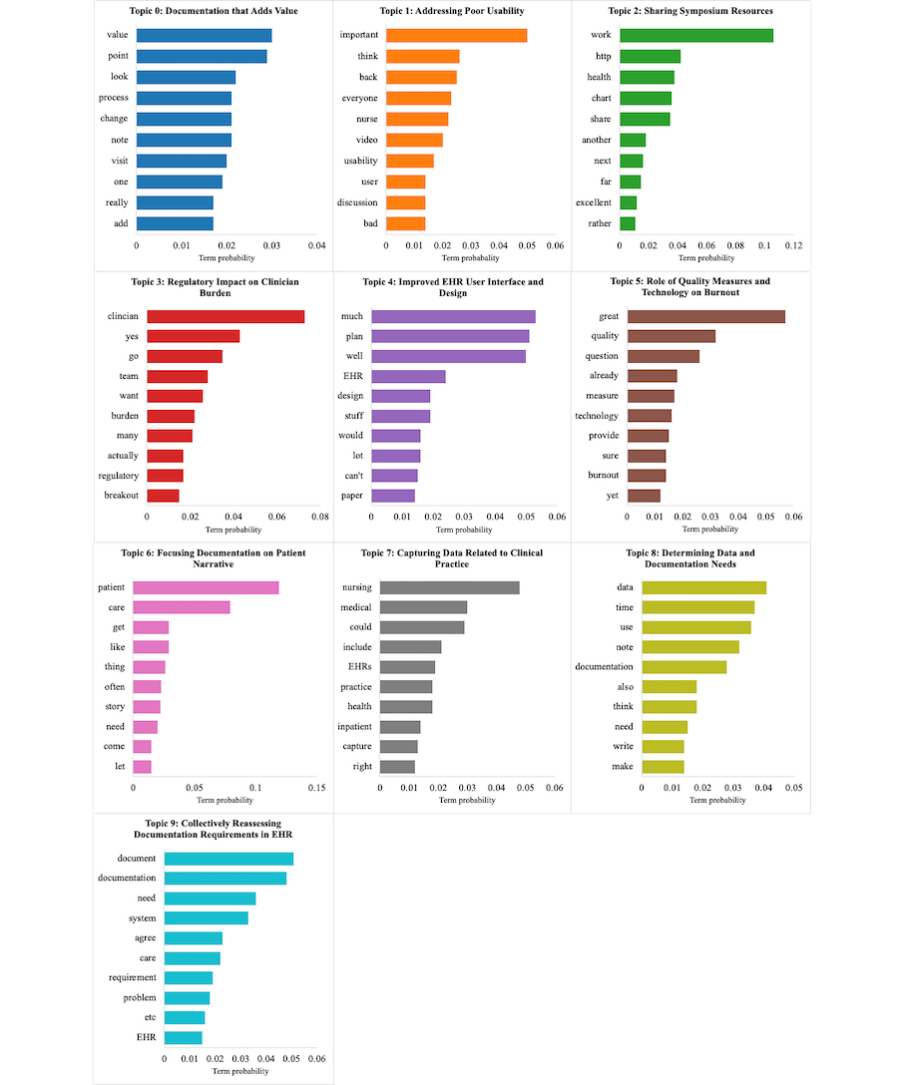
Top 10 term probabilities for each of the 10 latent Dirichlet allocation (LDA) model–identified topics. EHR: electronic health record.

The optimal LDA model (*C_v*=0.44) yielded 10 topics (Table 2). The top 10 term probabilities for these 10 topics are visualized in Figure 3. Among those 10 topics, *determining data and documentation needs* (422/1773, 23.8%) occurred at the highest frequency, followed by *collectively reassessing documentation requirements in EHRs* (252/1773, 14.2%) and *focusing documentation on patient narrative* (162/1773, 9.1%).

**Figure 4:**
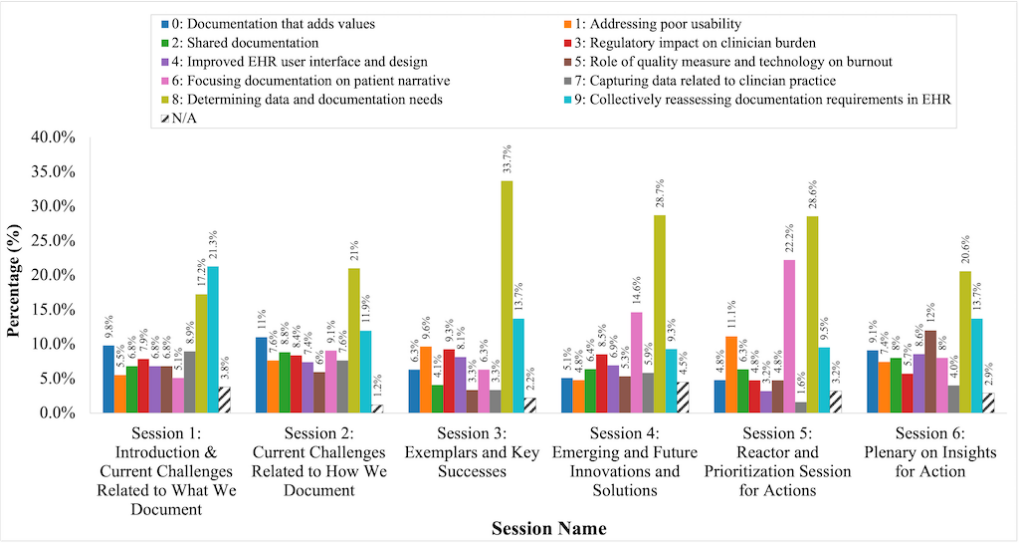
Distribution of the 10 latent Dirichlet allocation (LDA) model–identified topics stratified by symposium session number. EHR: electronic health record; N/A: not applicable.

**Figure 5:**
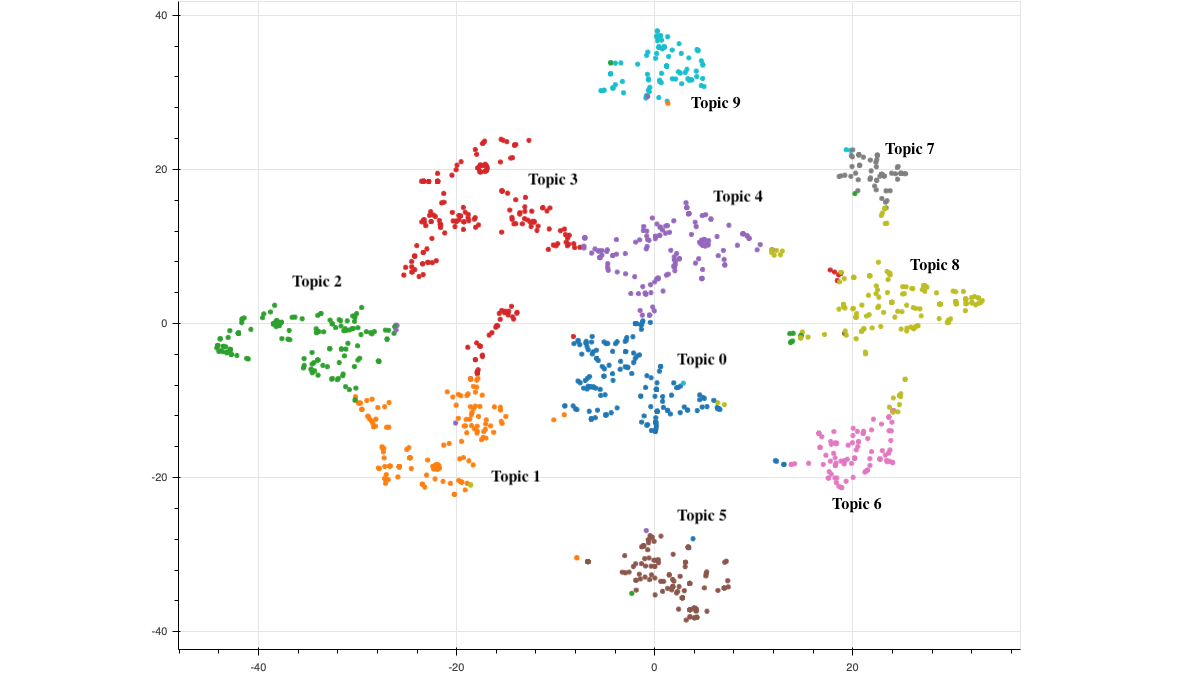
t-distributed Stochastic Neighbor Embedding (t-SNE) plot visualizing the 10 latent Dirichlet allocation (LDA) model–identified topics among the symposium chat logs.

Determining data and documentation needs had the highest prevalence throughout all sessions, between 17.2% (81/470) and 33.7% (91/270), except for Session 1 (Introduction & Current Challenges Related to What We Document), wherein collectively reassessing documentation requirements in EHRs represented the largest proportion (100/470, 21.3%) (Figure 4). The t-SNE plot demonstrated low overlap between topic clusters (Figure 5).

While not heavily represented in other sessions, *focusing documentation on patient narrative* had the second highest proportions in both Session 4 (Emerging and Future Innovations and Solutions) and Session 5 (Reactor and Prioritization Session for Actions). Among these 10 topics, 5 high-level thematic categories emerged: *consensus building* (821/1773, 46.3%)*, burden sources* (365/1773, 20.6%)*, EHR design* (250/1773, 14.1%)*, patient-centered care* (162/1773, 9.1%)*,* and *symposium highlights* (122/1773, 6.9%).

**Table 1:** Descriptive statistics on raw chat messages across all 6 symposium sessions.

Session number	Session title	Total attendees, N	Unique chat participants, n^a^ (%)	Chat message frequency, n^b^ (%)	Messages per participant, mean (SD)	Words per chat message			
						Mean (SD)	Min^c^	Med^d^	Max^e^
1	Introduction & Current Challenges Related to What We Document	203	99 (48.8)	470 (26.5)	4.7 (5)	18.4 (16.2)	1	14	108
2	Current Challenges Related to How We Document	185	81 (43.8)	419 (23.6)	5.2 (6.3)	18 (13.3)	1	15	51
3	Exemplars and Key Successes	169	52 (30.8)	270 (15.2)	5.2 (5.8)	21.8 (15.3)	1	19	51
4	Emerging and Future Innovations and Solutions	173	65 (37.6)	376 (21.2)	5.8 (6.2)	22.5 (21.2)	1	17	122
5	Reactor and Prioritization Session for Actions	N/A^f^	33 (n/a)	63 (3.6)	1.9 (1.5)	15 (11.9)	1	12	58
6	Plenary on Insights for Action	N/A^f^	54 (n/a)	175 (9.9)	3.2 (2.8)	19.5 (19.1)	1	14	115

^a^Proportion of within-session attendees.

^b^Proportion of all messages across 6 sessions.

^c^Min: minimum.

^d^Med: median.

^e^Max: maximum.

^f^N/A: not applicable. Data were not captured due to the breakout session format.

**Table 2:** Distribution of topics identified with raw example text among the symposium chat logs.

Topic identification number/label^a^	Thematic category	Raw example text^b^	Overall frequency, n (%)
0: Documentation that adds value	Consensus building	“It can be templated, it probably shouldn’t be documented. Low value note bloat relates to smart phrases and templates.” “The main point seems to be that we need to figure out what data adds value and getting rid of everything that does not.”	147 (8.3)
1: Addressing poor usability	EHR^c^ design	“I don't think linearly enough to dictate efficiently.” “User centered design is SOOOO important. At [medical center] over a 6 month period [EHR vendor] users were presented with [number] alerts, of which [number] interrupted their workflow (‘popped up’). Only 12% had any action taken‚ an 88% override rate.”	122 (6.9)
2: Sharing symposium resources	Symposium comments	“[Proper noun] you may be kindred spirits with [proper noun]‚ ‘Sloppy and Paste’ [URL].” “Are you sharing the 25X5 Zoom background? :)”	122 (6.9)
3: Regulatory impact on clinician burden	Burden sources	“Hard to hit the target when there are 6 divergent targets.” “[proper noun] Yes - focused on US Clinicians, given several problems related to doc burden are unique to US clinicians/US healthsystem.”	142 (8)
4: Improved EHR user interface and design	EHR design	“Better EHR design would allow it to be more integrated into the documentation workflow.” “Paper wins on portability!!”	128 (7.2)
5: Role of quality measures and technology on burnout	Burden sources	“My favorite mis-dictation: a person with a prosthetic valve: ‘poor sign valve.’” “The question is how can technology augment the cognition of the clinician.”	110 (6.2)
6: Focusing documentation on patient narrative	Patient-centered care	“Prime reason for using handheld devices in the exam room - so you can interact with the patient.” “Patient engagement in problem list reconciliation needed as patients move across encounters and care settings like advanced hospital care in the home.”	162 (9.1)
7: Capturing data related to clinical practice	Burden sources	“Do the problems in the USCDI v2 include nursing problems or are they only medical problems?” “[Proper noun] and the documenting patient valuables have contribution nothing to nursing practice or outcome.. but the risk manager wants it to remain. Sigh.”	113 (6.4)
8: Determining data and documentation needs	Consensus building	“The problem list is relatively useless since problems are never resolved making it difficult to slog through and determining what is truly an active problem.” “[Proper noun] - all the time. The problem (one of them at least) is that we have the write the same information in so many diff places. So there are naturally contradictions because we cant Keep it ALL updated.”	422 (23.8)
9: Collectively reassessing documentation requirements in EHRs	Consensus building	“Aligning documentation requirements key for safe care transitions, e.g. the [proper noun] project.” “Would be good to standardize documentation aimed at regulatory/acced requirements and have the agencies vet what is actually required.”	252 (14.2)
N/A^d,e^	N/A	N/A	53 (3)

^a^Numbering is based on the indices of an array to be consistent with programming code used across algorithms, which initiates with 0.

^b^Raw data are the actual chat messages of symposium attendees and have not been corrected for grammar.

^c^EHR: electronic health record.

^d^N/A: not applicable.

^e^Exclusively comprised of person names, stop words, and other terms removed at the preprocessing stage.

## Discussion

### Principal Findings

Traditionally, qualitative interviews have been applied to understand the clinician documentation burden [18]. To our best knowledge, no studies have computationally examined chat logs from a web-based symposium setting involving multiple participants or explicitly identified clinician documentation burden themes in chat logs using topic modeling. Based on our results, LDA may be a feasible approach to rapidly extracting high-level, semantically meaningful information generated in chat logs (an unstructured format) and detecting themes that may be of importance among participants, such as those surrounding documentation burden.

We conducted an exploratory analysis using 25X5 Symposium chat logs to elicit additional insights and context on documentation burden. Not surprisingly, 6 terms were particularly prominent based on the distribution of terms used among 25X5 Symposium participants who engaged in the chat: “need” (*rel_freq*=1), “patient” (*rel_freq*=0.94), “documentation” (*rel_freq*=0.92), and “data” (*rel_freq*=0.77), “note” (*rel_freq*=0.66), and “EHR” (*rel_freq*=0.61)] (Figure 2). Among the 10 topics that our LDA model identified, the top 4 most-referenced topics cumulatively represented more than half the chat messages; these topics included (1) *determining data and documentation needs*; (2) *collectively reassessing documentation requirements in EHRs;* (3) *focusing documentation on patient narrative*; and (4) *documentation that adds value* (Table 2). Notably, these topics represent 2 distinct high-level thematic categories that highlight future directions and prerequisites to alleviating documentation burden: *consensus building* (ie, evaluating the existing state of excessive and/or extraneous documentation including value-added documentation, data and documentation needs, and requirements imposed on EHRs) and *patient-centered care* (ie, focusing documentation on the synthesis of the patient’s story)*,* respectively. Another 2 topics, *collectively reassessing documentation requirements in EHRs* and *determining data and documentation needs*, co-occurred at the highest volume in all sessions with the exception of Session 4 (Emerging and Future Innovations and Solutions) and Session 5 (Reactor and Prioritization Session for Actions). Interestingly, *focusing documentation on patient narrative* and *determining data and documentation needs* emerged in Sessions 4 and 5 as the top 2 steps to address clinician documentation burden; this finding suggests that clinician documentation focusing on the patient’s story may be perceived as less burdensome—a view that may be supported by numerous stakeholders in addition to clinicians.

It is worthwhile to note that while topics that characterized the causes of burden such as *regulatory impact on clinician burden, capturing data related to clinician practice*, *role of quality measures and technology on burnout, improved EHR user interface and design,* and *addressing poor usability* appeared less frequently, it does not reflect each topic’s overall importance. For example, these topics were consistent with 5 of 6 documentation burden domains that the American Nursing Informatics Association (ANIA) identified (ie, “regulatory,” “self-imposed,” “quality,” “interoperability,” and “usability”). This was a key conceptual framework applied in the 25X5 Symposium [46], and it speaks to the validity of our model’s findings. While the ANIA-identified “reimbursement” domain did not emerge as a dominant topic from the 25X5 Symposium chat log content, “cms” (ie, Centers for Medicare and Medicaid Services; *rel_freq*=0.16) and “billing” (*rel_freq*=0.15) were among the top 100 most frequently used terms throughout the 25X5 Symposium chats. Thus, these latent topics identified among symposium participants may additionally represent salient future directions that should be further assessed and prioritized for policy and practice.

Our topic modeling analysis uncovered themes associated with two parallel processes that emerged over the web-based symposium platform: (1) unstructured conversations regarding the clinician documentation burden previously described above, and (2) discussions focused on the content and format of the presentations, such as *sharing the 25X5 Symposium resources* (Table 1). Because attendees were encouraged to engage in the chat as they personally saw fit, chat discussion topics were unstructured and emerged organically, which may or may not have been pertinent to the concurrent presentations that were being held. This format generated themes that were highly heterogeneous—for example, content-, opinion-, or administrative-related comments. However, this phenomenon is not unique to the 25X5 Symposium, as “distraction and division of attention” [22] have been identified as a potential shortcoming to web-based meetings with synchronous chat functionalities made available; synchronous chats may provide a platform for impertinent topics to emerge and become a source of distraction [22]. Additionally, because synchronous chats are dynamic, extracting high-quality topics from chat logs may be difficult, as topics continually change and evolve longitudinally without the same conversational constraints as spoken language [47]. Nevertheless, chat content presents an opportunity to understand participant sentiment on the content areas presented (eg, clinician documentation burden) as well as the operational aspects of the symposium. To structure chat content analyses and adjust the granularity of topics identified, future web-based symposia may consider incorporating interactive prompts throughout presentations in the chat to engage participants in specific thematic areas. Computationally, topic-oriented ranking with context-aware autoencoders, such as Bidirectional Encoder Representations from Transformers (BERT) may be an approach to improve topic model analyses of documents (eg, chat logs) with rapidly evolving, fragmented topics [48]. Finally, knowledge of participant demographics (eg, employment, specialty areas, age) may facilitate high-resolution network analyses of participant interactions and their level of importance [49].

Our initial aim was to investigate the distribution of topics over time in parallel with transcripts generated from the presentation content. Although 1000 documents were identified as adequate for conducting topic modeling [50], we did not have a sufficient volume (approximately 1800 documents) to examine term-probability distributions and topics in 1-minute intervals. Additionally, parsing presentation transcripts into 1-minute intervals did not make sense as each speaker presentation (approximately 15 minutes long) logically represents 1 document; thus, these documents do not exist on the same scale. Future research focused on comparative analyses of parallel chat and presentation content among web-based symposia may find success with shorter presentations that occur at high volume, where a set of chat messages—like presentation content—may be treated as 1 document and therefore exist on the same scale.

Furthermore, prior topic modeling applications suggest documents should be, at minimum, 3 sentences long [50]. While high-volume Twitter data have been previously examined using LDA models [51], topic models on short textual content such as those represented in the 25X5 Symposium chat messages—which were, on average, approximately 20 words in length (ie, roughly 1 sentence long)—coupled with low document volume, tend to yield sparse and noisy results. Due to the reduced likelihood of terms co-occurring among these types of collections of documents, repetitive or low-quality topics may have emerged [42,52]. Nevertheless, the lack of crossover among topic clusters as identified in our t-SNE plot indicate themes of high quality (Figure 5). Additionally, we could not qualitatively identify a notable correlation between the distribution of the topics discussed in the chat messages (Figure 4) and the content of the speaker presentations (available on the 25X5 Symposium website [16]) at a high level [53]. However, we can distinguish that the chat content from Sessions 1 to 3 (which focused on the existing documentation landscape) emphasized topics such as *collectively reassessing documentation requirements in EHRs* and *determining data and documentation needs* (ie, the current state), while chat content from Sessions 4 and 5 (which centered around future directions) underscored topics including *focusing documentation on patient narrative* and *determining data and documentation needs* (ie, the future state). Therefore, chat logs may provide additional valuable contextual information on the receptiveness and priorities of attendees on the content presented in web-based formats in an unobtrusive and fluid fashion—and in this scenario, clinician documentation burden.

In this analysis, we note that we iterated over the chat logs to remove unique identifiers including person names and Zoom user handles using a rule-based approach (ie, regular expressions) supplemented with manual human annotation. Among the raw corpus, approximately 6.5% (n=270) of the terms represented person names; this was anticipated as attendee chat interactions were impersonal and fluid and frequently referred to fellow attendee messages. Chat log content is distinctive because Zoom user handles are theoretically and infinitely unique. Programming techniques such as employing “chunking” to extract terms that are part-of-speech tagged as proper nouns (ie, “NNP”) are imperfect and indiscriminate. For example, “chunking” was unable to detect Zoom user handles satisfactorily and resulted in data loss among important proper nouns (ie, those not associated with person names) that we sought to retain in the analysis such as “the joint commission,” “tjc” (ie, the Joint Commission), and “cms” to uncover more meaningful topics. Thus, trade-offs exist in how researchers opt to preprocess chat log data. While our application of topic modeling on chat logs is novel, this challenge has been documented and similarly discussed among well-known, large clinical datasets such as the Beth Israel Deaconess Medical Center data via the Medical Information Mart for Intensive Care III (MIMIC-III), which employ manual, rule-based methods (eg, pattern-matching with regular expressions and dictionary lookups) to deidentify textual data [54]. As mentioned previously, such techniques for deidentifying clinical textual data frequently overlook unconventional proper nouns and other edge cases. Future efforts should explore more advanced and generalizable privacy-protective methods for deidentifying unstructured textual data, particularly in chat logs [54].

### Limitations

As with all secondary analyses, this study has several limitations. As attendees who participated in the 25X5 Symposium, and those who engaged with the synchronous chat functionality may not be representative of all clinicians and health care leaders, selection bias may be present in the data. Furthermore, it is possible that some attendees experienced reluctance in sharing their comments and opinions in a public forum (ie, Hawthorne effect) [12], which would be memorialized in perpetuity. However, prior evidence on video conference meetings with parallel chat functionalities [22] indicates that synchronous chats foment inclusivity in engagement among those who would otherwise not have an opportunity to contribute their thoughts (eg, more introverted participants) or prefer alternative communication modalities (eg, written language). Overall, there was no evidence that suggested participants felt uncomfortable sharing their thoughts during the 25X5 Symposium.

Additionally, there was marginal attendee attrition from Session 1 (n=203) to Session 4 (n=173); however, new participants joined as previous participants exited, and we were unable to verify which participants attended Sessions 5 and 6. Overall, over 70% (n=209) of attendees joined more than 1 session between Sessions 1 and 4. Fewer attendees participated in the chat functionality in Sessions 5 and 6 as compared to Session 1. However, this is fitting, as Sessions 5 and 6 were reactor sessions and participants were more engaged in the private breakout discussion sections that did not include access to the chat functionality. Alternatively, Session 1 was the first instance many attendees had convened in such a large group during the COVID-19 pandemic to interactively discuss clinician documentation burden. These factors may have stimulated discussion early on. Nevertheless, as we did not disseminate an exit survey, we were unable to ascertain why some participants “dropped out.” Finally, between-participant chat messages were not captured in Zoom. As a result, the subsequent topics related to documentation burden identified in this analysis may not be exhaustive of all themes that concern the greater population. Therefore, our results may not be generalizable to settings outside of the 25X5 Symposium.

### Future Directions

As we continue to refine our models and explore network analyses, additional results will be forthcoming. We have already generated short-, medium- and long-term goals to reduce documentation burden immediately following the 25X5 Symposium [16,55]. Future efforts will focus on determining how to highlight and prioritize the themes identified in this study to ensure that they represent concrete and actionable focus areas and recommendations within the 25X5 documentation reduction framework. Presently, the 25X5 Symposium’s objectives continue to persist nationwide through the AMIA 25X5 initiative [53], which has been harnessing key stakeholder expertise to investigate how to best evaluate documentation burden [56], streamline workflows, and optimize EHRs.

Given the ongoing challenges of recruiting clinicians for qualitative studies post pandemic, topic modeling may offer an alternative and less intrusive approach to investigating clinician documentation burden and burnout using their own words. For instance, topic modeling analysis of high-volume textual data sources not unconventionally applied in this domain, such as Twitter data, may provide timely and relevant representations of documentation burden themes among clinicians, health care leaders, and other stakeholders at any given point in time or facilitate the monitoring of clinician sentiment and areas of interest over time. Alternatively, there is a potential for topic modeling to be applied to longitudinally investigate documentation burden by examining the evolution of clinical note content. Consequently, topic modeling may complement existing mixed methods research.

### Conclusions

In this study, we employed topic modeling on unstructured textual content from the 25X5 Symposium to explore the feasibility of this novel application to multiparticipant chat logs and elicit additional insights on clinician documentation burden from the web-based symposium. Our findings uncovered 4 critical high-level areas to consider when resolving clinician documentation burden: achieving consensus on existing and future interventions; identifying specific causes of burden; refining EHR design, user interface, and usability; and improving the synthesis of the patient narrative. Topic modeling may be a valuable method to rapidly examine latent themes presented in chat logs as well as unobtrusively investigate topics associated with clinician documentation burden using unstructured textual content.

## Abbreviations

25X5 Symposium: 25 by 5: Symposium to Reduce Documentation Burden on US Clinicians by 75%
AMIA: American Medical Information Association
ANIA: American Nursing Informatics Association
BERT: Bidirectional Encoder Representations From Transformers
EHR: electronic health record
LDA: latent Dirichlet allocation
MIMIC-III: Medical Information Mart for Intensive Care III
t-SNE: t-distributed Stochastic Neighbor Embedding

## References

[1] National Academies of Sciences, Engineering, and Medicine, National Academy of Medicine, Committee on Systems Approaches to Improve Patient Care by Supporting Clinician Well-Beingv (2019). Taking Action Against Clinician Burnout: A Systems Approach to Professional Well-Being.

[2] Reducing administrative burden. American Medical Association.

[3] Strategy on reducing regulatory and administrative burden relating to the use of health IT and EHRs. Department of Health and Human Services Office of the National Coordinator for Health Information Technology.

[4] Verma S (2020). Patients over paperwork. US Department of Health & Human Services.

[5] Hatmaker D Re: Strategy on reducing regulatory and administrative burden relating to the use of health IT and EHRs draft report. American Nurses Association.

[6] Hull S, Mitchell MB (2019). Comments on draft strategy to reduce documentation burden. Alliance for Nursing Informatics.

[7] Reducing clinician burden. Health Level 7 International.

[8] Cohen G, Brown L, Fitzgerald M, Somplasky A (2019). Exploring the feasibility of using audit log data to quantitate burden as providers use electronic health records. Mathematica.

[9] Colicchio TK, Cimino JJ, Del Fiol G (2019). Unintended consequences of nationwide electronic health record adoption: challenges and opportunities in the post-meaningful use era. J Med Internet Res.

[10] Padden J (2019). Documentation burden and cognitive burden: how much is too much information?. Comput Inform Nurs.

[11] Downing NL, Bates DW, Longhurst CA (2018). Physician burnout in the electronic health record era: are we ignoring the real cause?. Ann Intern Med.

[12] Sinsky C, Colligan L, Li L, Prgomet M, Reynolds S, Goeders L, Westbrook J, Tutty M, Blike G (2016). Allocation of physician time in ambulatory practice: a time and motion study in 4 specialties. Ann Intern Med.

[13] Arndt BG, Beasley JW, Watkinson MD, Temte JL, Tuan W, Sinsky CA, Gilchrist VJ (2017). Tethered to the EHR: primary care physician workload assessment using EHR Event log data and time-motion observations. Ann Fam Med.

[14] Frogner BK, Dill JS (2022). Tracking turnover among health care workers during the COVID-19 pandemic: a cross-sectional study. JAMA Health Forum.

[15] Sinsky CA, Brown RL, Stillman MJ, Linzer M (2021). COVID-related stress and work intentions in a sample of US health care workers. Mayo Clin Proc Innov Qual Outcomes.

[16] 25 x 5 Symposium drives ongoing efforts to reduce documentation burden on U.S. clinicians. Columbia University Department of Biomedical Informatics.

[17] Holmgren AJ, Downing NL, Bates DW, Shanafelt TD, Milstein A, Sharp CD, Cutler DM, Huckman RS, Schulman KA (2021). Assessment of electronic health record use between US and non-US health systems. JAMA Intern Med.

[18] Rowlands S, Tariq A, Coverdale S, Walker S, Wood M (2022). A qualitative investigation into clinical documentation: why do clinicians document the way they do?. Health Inf Manag.

[19] Remmel A (2021). Scientists want virtual meetings to stay after the COVID pandemic. Nature.

[20] Kim K, Kim SR, Lee J, Moon J, Lee S, Shin SJ (2022). Virtual conference participant's perceptions of its effectiveness and future projections. BMC Med Educ.

[21] Chen X, Wang Y (2004). Use synchronous chat to improve online learning experience.

[22] Sarkar A, Rintel S, Borowiec D, Bergmann R, Gillett S, Bragg D, Baym N, Sellen A (2021). The promise and peril of parallel chat in video meetings for work.

[23] Mak B, Chui H (2015). Learning through instant-messaging chat logs: a tool for adults to address the communication norms in the new workplace. Emerging Issues in Smart Learning. Lecture Notes in Educational Technology.

[24] Holmer T (2008). Discourse structure analysis off chat communication. Language@Internet.

[25] Park K, Kim J, Park J, Cha M, Nam J, Yoon S, Rhim E (2015). Mining the minds of customers from online chat logs. https://dl.acm.org/doi/proceedings/10.1145/2806416.

[26] Wang T, Huang Z, Gan C (2016). On mining latent topics from healthcare chat logs. J Biomed Inform.

[27] Liu L, Tang L, Dong W, Yao S, Zhou W (2016). An overview of topic modeling and its current applications in bioinformatics. Springerplus.

[28] Zengul F, Lee T, Delen D, Almehmi A, Ivankova N, Mehta T, Topuz K (2020). Research themes and trends in ten top-ranked nephrology journals: a text mining analysis. Am J Nephrol.

[29] Mokkenstorm JK, Eikelenboom M, Huisman A, Wiebenga J, Gilissen R, Kerkhof AJFM, Smit JH (2017). Evaluation of the 113Online suicide prevention crisis chat service: outcomes, helper behaviors and comparison to telephone hotlines. Suicide Life Threat Behav.

[30] Ahlström Britt H, Wentz E (2014). Difficulties in everyday life: young persons with attention-deficit/hyperactivity disorder and autism spectrum disorders perspectives. A chat-log analysis. Int J Qual Stud Health Well-being.

[31] Chen X, Wang H (2019). Automated chat transcript analysis using topic modeling for library reference services. Proceedings of the Association for Information Science and Technology.

[32] Hristova G (2020). Topic modeling of chat data: a case study in the banking domain. Conference Proceedings of the American Institute of Physics.

[33] Denton CA, Soni HC, Kannampallil TG, Serrichio A, Shapiro JS, Traub SJ, Patel VL (2018). Emergency physicians' perceived influence of EHR use on clinical workflow and performance metrics. Appl Clin Inform.

[34] Moy AJ, Schwartz JM, Withall J, Lucas E, Cato KD, Rosenbloom ST, Johnson K, Murphy J, Detmer DE, Rossetti SC (2021). Clinician and health care leaders' experiences with-and perceptions of-COVID-19 documentation reduction policies and practices. Appl Clin Inform.

[35] Gaffney A, Woolhandler S, Cai C, Bor D, Himmelstein J, McCormick D, Himmelstein DU (2022). Medical documentation burden among US office-based physicians in 2019: a national study. JAMA Intern Med.

[36] Moy AJ, Hobensack M, Marshall K, Vawdrey D, Kim E, Cato K, Rossetti S (2023). Understanding the perceived role of electronic health records and workflow fragmentation on clinician documentation burden in emergency departments. J Am Med Inform Assoc.

[37] Rehurek R, Sojka P (2011). Gensim–python framework for vector space modelling. NLP Centre, Faculty of Informatics, Masaryk University, Brno, Czech Republic.

[38] Bird S, Klein E, Loper E (2009). Natural Language Processing with Python: Analyzing Text with the Natural Language Toolkit.

[39] Naveed N, Gottron T, Kunegis J, Che AA (2011). Searching microblogs: coping with sparsity and document quality.

[40] Blei D, Ng A, Jordan M (2003). Latent dirichlet allocation. J Mach Learn Res.

[41] Porturas T, Taylor RA (2021). Forty years of emergency medicine research: Uncovering research themes and trends through topic modeling. Am J Emerg Med.

[42] Albalawi R, Yeap TH, Benyoucef M (2020). Using topic modeling methods for short-text data: a comparative analysis. Front Artif Intell.

[43] O’Callaghan D, Greene D, Carthy J, Cunningham P (2015). An analysis of the coherence of descriptors in topic modeling. Expert Syst Appl.

[44] Van der Maaten L, Hinton G (2008). Visualizing data using t-SNE. J Mach Learn Res.

[45] Kadayat B, Eika E (2020). Impact of sentence length on the readability of web for screen reader users.

[46] Sengstack P, Adrian B, David R, Boyd L, Davis A, Hook M, Hulett S, Karp E, Kennedy R, Heermann L, Niblett T (2020). The six domains of burden: a conceptual framework to address the burden of documentation in the electronic health record. Position paper of the American Nursing Informatics Association Board of Directors. American Nursing Informatics Association.

[47] Uthus D, Aha D (2013). Multiparticipant chat analysis: A survey. Artif Intell.

[48] Zou Y, Lin J, Zhao L, Kang Y, Jiang Z, Sun C, Zhang Q, Huang X, Liu X (2020). Unsupervised summarization for chat logs with topic-oriented ranking and context-aware auto-encoders.

[49] Saqr M, Nouri J (2020). High resolution temporal network analysis to understand and improve collaborative learning.

[50] Topic modeling. Amazon Web Services.

[51] Delir Haghighi P, Burstein F, Urquhart D, Cicuttini F, Robert (2021). Investigating individuals' perceptions regarding the context around the low back pain experience: topic modeling analysis of Twitter data. J Med Internet Res.

[52] Wu X, Li C, Zhu Y, Miao Y (2020). Short text topic modeling with topic distribution quantization and negative sampling decoder.

[53] Levy DR, Sloss EA, Chartash D, Corley ST, Mishuris RG, Rosenbloom ST, Tiase VL (2023). Reflections on the documentation burden reduction AMIA plenary session through the lens of 25 × 5. Appl Clin Inform.

[54] Ahmed T, Aziz MA, Mohammed N (2020). De-identification of electronic health record using neural network. Sci Rep.

[55] Hobensack M, Levy DR, Cato K, Detmer DE, Johnson KB, Williamson J, Murphy J, Moy A, Withall J, Lee R, Rossetti SC, Rosenbloom ST (2022). 25 × 5 Symposium to Reduce Documentation Burden: report-out and call for action. Appl Clin Inform.

[56] Moy A, Schwartz J, Chen R, Sadri S, Lucas E, Cato K, Rossetti S (2021). Measurement of clinical documentation burden among physicians and nurses using electronic health records: a scoping review. J Am Med Inform Assoc.

